# Preparedness for Threat of Chikungunya in the Pacific

**DOI:** 10.3201/eid2008.130696

**Published:** 2014-08

**Authors:** Adam Roth, Damian Hoy, Paul F. Horwood, Berry Ropa, Thane Hancock, Laurent Guillaumot, Keith Rickart, Pascal Frison, Boris Pavlin, Yvan Souares

**Affiliations:** Secretariat of the Pacific Community, Noumea, New Caledonia (A. Roth, D. Hoy, P. Frison, Y. Souares);; Papua New Guinea Institute of Medical Research, Goroka, Papua New Guinea (P.F. Horwood);; National Department of Health, Port Moresby, Papua New Guinea (B. Ropa);; Yap State Department of Health Services, Federated States of Micronesia (T. Hancock);; Institut Pasteur, Noumea (L. Guillaumot);; Queensland Health, Brisbane, Queensland, Australia (K. Rickart);; World Health Organization, Port Moresby (B. Pavlin)

**Keywords:** chikungunya, CHIKV, Pacific, Pacific Public Health Surveillance Network, PPHSN, Aedes aegypti, Aedes albopictus, outbreak, preparedness, viruses, vector-borne infections, mosquitos, Indian Ocean, Papua New Guinea, Yap State, Federated States of Micronesia, New Caledonia

## Abstract

Chikungunya virus (CHIKV) caused significant outbreaks of illness during 2005–2007 in the Indian Ocean region. Chikungunya outbreaks have also occurred in the Pacific region, including in Papua New Guinea in 2012; New Caledonia in April 2013; and Yap State, Federated States of Micronesia, in August 2013. CHIKV is a threat in the Pacific, and the risk for further spread is high, given several similarities between the Pacific and Indian Ocean chikungunya outbreaks. Island health care systems have difficulties coping with high caseloads, which highlights the need for early multidisciplinary preparedness. The Pacific Public Health Surveillance Network has developed several strategies focusing on surveillance, case management, vector control, laboratory confirmation, and communication. The management of this CHIKV threat will likely have broad implications for global public health.

Chikungunya virus (CHIKV) is an alphavirus transmitted to humans by *Aedes* species mosquitoes, particularly *Aedes aegypti* and *A. albopictus* (*1*). It typically causes fever and severe and persistent joint pain (*2*). CHIKV was first recognized as a human pathogen in 1952 in Tanzania and, after several decades of little activity, has reemerged globally during the past decade (*3*). Chikungunya first appeared in the Pacific region in a small outbreak in New Caledonia in 2011 (*1*), but the virus is now a major threat in this reason. Outbreaks have been confirmed in Papua New Guinea (PNG) in June 2012 (*4*); New Caledonia in April 2013 (*5*); and Yap State, Federated States of Micronesia, in August 2013 (*6*). In this article, we give an overview of the virus, update the recent epidemiology of CHIKV, and assess the risk for CHIKV spread in the Pacific. We draw on lessons learned from the response efforts in the Indian Ocean, where the most devastating chikungunya epidemic so far caused havoc in a setting that is very similar to that of the Pacific Islands (*7*). We propose a series of public and clinical health measures to help Pacific Island countries and territories prepare for potential outbreaks of CHIKV infection.

## Recent and Current Epidemiology of Chikungunya

Since 2000, chikungunya outbreaks have occurred in several regions of the world (*3*). Broadly speaking, the locations of these outbreaks appear to be moving in an easterly direction (*3*). Outbreaks occurred in the Democratic Republic of Congo in 2000 (*8*) and in Indonesia in 2001–2003 (*9*). In 2004, the virus appeared in Kenya; subsequently, a series of outbreaks occurred in the Indian Ocean during 2005–2007 (*10*). Affected locations included Seychelles (≈9,000 cases) (*11*), Comoros Islands (including 215,000 cases on Grande Comore) (*12*), Madagascar (*12*), Reunion Island (266,000 cases and 250 deaths) (*12*), Mauritius (≈6,000 cases) (*11*), and the Republic of the Maldives (*13*). During 2006–2007, outbreaks occurred in South and Southeast Asia (*14*). India reported 1.4 million cases (*15*), Sri Lanka 37,667 cases (*15*), and Malaysia 200 cases (*15*). Cases were also reported in Singapore in 2008 (*16*) and Thailand in 2008–2009 (*17*). The outbreaks in the Indian Ocean resulted in high attack rates, for example, 63% of the population in Grande Comore and 35% in Reunion Island (*3*,*14*,*18*).

Local chikungunya transmission had not been reported in the Pacific region until February 2011, when an autochthonous transmission of CHIKV was reported in New Caledonia (*19*). The first 2 cases were in persons who had recently returned from Indonesia; consistently, the virus was shown to belong to the Asian lineage. Only 33 cases were detected in total, and the outbreak was halted through aggressive case finding and vector control (*1*).

**Figure 1:**
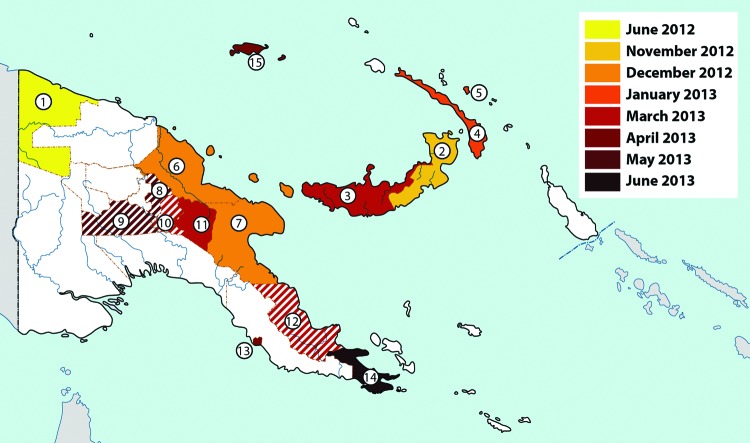
Chikungunya epidemic in Papua New Guinea, 2012–2013. Colors denote the time for reports or rumors of emerging clinical disease. When such information was lacking, the date of laboratory confirmation of chikungunya virus infection determined the color coding. Solid colors indicate that cases were laboratory confirmed; striped colors indicate lack of laboratory confirmation.

In June 2012, a chikungunya outbreak started in West Sepik Province of PNG (*20*). In December, the PNG National Department of Health reported on PacNet, which is the Pacific Public Health Surveillance Network (PPHSN) early warning system (*21*), that similar but unconfirmed cases had been detected in Madang and East New Britain Provinces. Since then, investigations have made it apparent that CHIKV spread east through PNG (Figure 1; Table 1) (*4*,*22*). In January 2013, confirmation of a case imported to Queensland, Australia, from PNG was reported (*23*). Since then, 10 more cases of chikungunya imported from PNG to Queensland have been reported (*24*).

**Table 1:** Spread of chikungunya epidemic based on case reports, Papua New Guinea, 2012–2013*

Province	Date reports or rumors of cases occurred	Date cases laboratory confirmed	Total no. cases
West Sepik	2012 Jun	2012 Oct	>1,500
East New Britain	2012 Nov	2013 Jan	>1,500
West New Britain		2013 Mar	
New Ireland	2013 Jan	2013 Jan	
Sanambiet Island (Lihir Island)	2013 Jan	2013 Jan	
Madang	2012 Dec	2013 Apr	
Morobe	2012 Dec	2013 Feb	
Jiwaka		2013 May	
Southern Highlands	2013 May		
Chimbu	2013 Mar	2013 Apr	
Eastern Highlands		2013 Mar	
Oro	2013 Mar		
National Capital District (Port Moresby)		2013 Apr	
Milne Bay	2013 Jun		
Manus	2013 Apr	2013 May	

*Blank cells indicate missing or incomplete data. For some provinces, only positive laboratory cases were reported without corresponding reports of cases and vice versa. Few provinces reported total number of cases.

At the end of April 2013, a chikungunya outbreak was confirmed in Noumea, the capital of New Caledonia (*5*). To date, 30 autochthonous cases have been confirmed, dating from early February through November 2013. The putative index case originated from the Indonesia, and the CHIKV was of the Asian lineage (data not shown).

In Yap State, an outbreak of chikungunya was reported in August 2013 (*6*). As of December 3, 2013, a total of 974 cases had been reported on Yap State and 128 cases on neighboring islands. The Yap State Department of Health Services posts weekly situational reports on PacNet to update the region. The path of introduction and the CHIKV genotype involved were not yet known at the time this article was prepared.

## Risk for Further Spread of CHIKV in the Pacific

**Figure 2:**
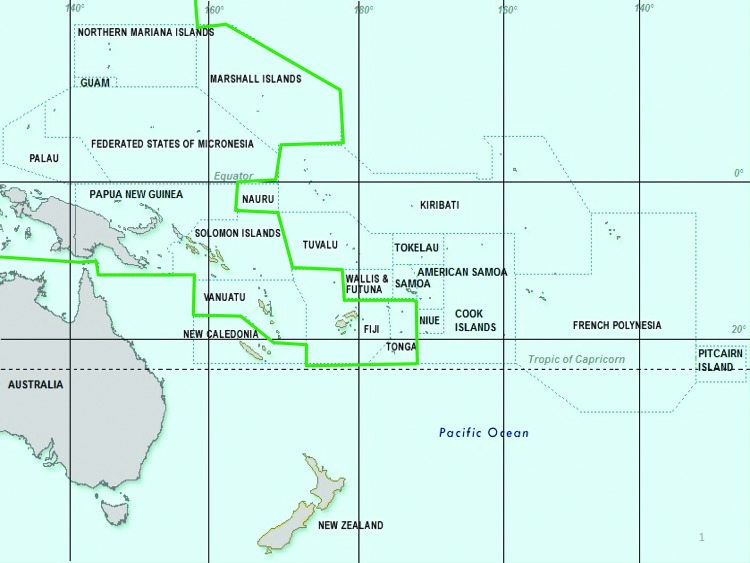
Distribution of chikungunya vectors *Aedes albopictus* and *Ae. aegypti* mosquitos in the Pacific, 2013. Green outline indicates areas where *Ae. albopictus* mosquitos are confirmed or strongly suspected. *Ae. aegypti* mosquitos are found in most locations except New Zealand, Hawaii, Futuna, and some remote islands. Dotted lines indicate member countries of Pacific Public Health Surveillance Network.

The CHIKV transmission cycle among humans can include *Ae. aegypti* or *Ae. albopictus* mosquitoes (*25*), both of which are widely spread in the Pacific region (Figure 2) (*26*). Some local *Aedes* mosquito species (e.g., *Ae. polynesiensis*) are also considered potential vectors. CHIKV has 3 genotypes, depending on its phylogenetic origins: West African, Asian, and East Central South African (ECSA) (*27*). The ECSA lineage may carry a point mutation in 1 gene of the E1 surface glycoprotein (E1:A226V), which greatly accelerates the replication cycle of the virus in the female *Ae. albopictus* mosquito, possibly from 5–7 days to 2–3 days (*25*). The ECSA virus strain was responsible for the largest documented epidemic of chikungunya in the Indian Ocean during 2005–2007 (*28*,*29*). CHIKV in PNG has been shown to be of the ECSA genotype and to carry this mutation (*4*). This genotype and mutation have also been confirmed in 4 of the recent cases imported to Queensland, Australia, from PNG (Forensic and Scientific Services, Department of Health, Queensland, Australia, pers. comm.). Furthermore, vector control measures in the Pacific may be hampered by pyrethroid resistance, which has already been described in *Ae. aegypti* mosquitoes in New Caledonia (*1*,*30*).

**Figure 3:**
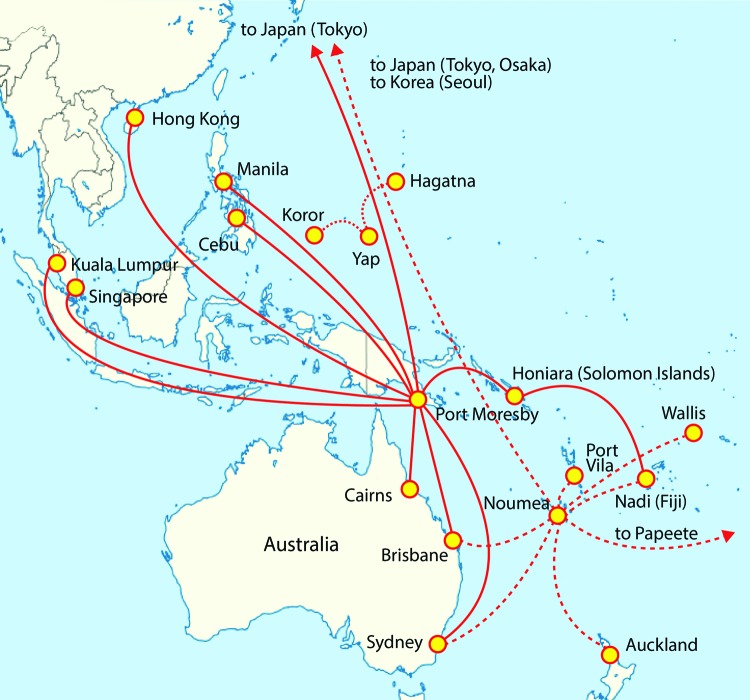
Direct airline routes to Pacific region destinations from Papua New Guinea (Port Moresby), New Caledonia (Noumea), and Yap State, Federated States of Micronesia.

Overall, the incidence of emerging diseases is increasing worldwide (*31*), partly because of population mobility and airline travel; >2 billion passengers take commercial flights every year (*32*). As demonstrated by outbreaks in Italy in 2007 and in France in 2010, both of which originated in India, CHIKV can spread by airline routes (*33*,*34*). Linking disease and vector distribution with air travel data is considered an important method for risk assessment of vector-borne disease spread (*32*). Considering the development of the situation in PNG, New Caledonia, and Yap State, the risk that cases of CHIKV infection will be imported to other Pacific Islands and is substantial, depending on travel patterns and numbers of airline passengers between countries and territories where CHIKV is circulating. The risk would be especially high for countries and territories with large numbers of air travelers to and from countries and territories with ongoing epidemics. Direct flights from PNG go to Fiji, Solomon Islands, Vanuatu, Australia, the Philippines, Hong Kong, Malaysia, Singapore, and Japan. From New Caledonia, direct flights go to Japan, South Korea, Australia, Fiji, Wallis Island, French Polynesia, and New Zealand; from Yap State, flights go to Guam and Palau (Figure 3).

Documentation on previous CHIKV circulation in the Pacific is scarce, but studies from PNG and Indonesia from the 1970s indicate a seroprevalence of CHIKV in the population of up to 30% (*35*,*36*). These results should be interpreted with caution because of known antigenic cross-reactivity of arboviruses, including CHIKV; however, these finding indicate that CHIKV could have circulated in the region and that there may be immunity among some populations.

Thus, similar to the situation in the Indian Ocean during the devastating chikungunya outbreaks in 2005–2007 (*7*), the risk of introduction of the virus to the Pacific region, followed by severe consequences for the area, is high because of virus strain, vector competence, and population mobility. The human population also likely has little or no immunity, making them susceptible to transmission, but this needs further study. However, differences between the Indian Ocean and the Pacific, particularly in population density, may decrease the risk of spread.

## Lessons Learned from Recent Chikungunya Outbreaks in the Indian Ocean

Chikungunya had not previously been reported in Reunion Island, but during March 2005–April 2007, a total of 266,000 people—about one third of the population—were infected with CHIKV, and ≈250 people died (*7*,*18*). This outbreak resulted in a tremendous burden on the health system, peaking with >47,000 estimated cases in 1 week (*7*). The main risk factors for complications and death from CHIKV infection were age >65 years and preexisting diabetes and cardiovascular diseases (*7*,*37*). The economic costs of the epidemic were extreme, in large part because of absenteeism among both patients and caregivers, and the island economy had to be rescued by the central government of France under a specific crisis funding mechanism (*38*). Recurring and chronic joint pain affected one third of patients for 3 months to 1 year, and some case-patients have had these symptoms even longer (*3*), so that they are still affecting the health system and the socioeconomic well-being of the island’s population (*38*,*39*). Outbreaks of chikungunya and other diseases can also have a negative effect on tourism (*40*,*41*); the tourism industry is therefore a potentially important stakeholder to engage in prevention work.

The chikungunya outbreak in Reunion Island highlighted the importance of using a multidisciplinary approach to address medical and public health issues (*42*). Numerous teams in the arbovirus community rapidly focused their studies on CHIKV. One noticeable initiative was the creation of a CHIKV task force, comprising virologists, epidemiologists, entomologists, pathologists, immunologists, and clinicians working in Reunion Island (*42*,*43*).

Several lessons were learned from this experience. First and foremost were the limitations of island health care systems, which focus mainly on primary health care, to cope with so many cases of severe illness (*44*). The outbreak also emphasized the need for early preparedness to ensure the following: removal of potential vector-breeding sites; strengthening of vector control teams; efficient case management; adequate surveillance, case detection, and information and communication strategies; and the development of clear and consistent messages for behavior change campaigns (*44*,*45*).

## Recommendations for Preparing for Chikungunya in the Pacific

To meet the need for early preparedness and consistent communication, the PPHSN has adopted an aggressive line of action in information dissemination. The PPHSN is a voluntary network of countries, territories, and organizations dedicated to the promotion of public health surveillance and appropriate response to the health emergencies for 22 Pacific Island countries and territories (Figure 2). The network was founded in 1996 under the auspices of the Secretariat of the Pacific Community and the World Health Organization (*21*).

The common surveillance system of the PPHSN is the Pacific Syndromic Surveillance System, which was introduced in October 2010 and implemented during the next 12 months in 20 of 22 Pacific Island countries and territories (*46*). This functional and timely regional infectious disease surveillance system tracks 4 core syndromes: acute fever and rash, diarrhea, influenza-like illness, and prolonged fever. Some countries also report the optional dengue-like illness. The frequency of syndromes is reported weekly to PPHSN partner World Health Organization in Suva, which prompts the countries and territories that there is an unexpected rise in a syndrome when the threshold of 90% of historically high reports is passed. The syndromes that would be expected to rise in frequency during dengue or chikungunya outbreaks are acute fever and rash, prolonged fever, and dengue-like illness (*46*,*47*). For confirmation testing of the causative agent of the outbreak, support is provided to Pacific Island countries and territories through the PPHSN laboratory referral network, LabNet (*21*).

In November 2012, a message was posted on PacNet relating that CHIKV infection occurred in PNG. In February 2013, a second communication was posted, relating to a case imported to Australia from PNG, including recommendations to the region (Table 2). The recommendations were derived through adapting international guidelines on preparedness for chikungunya outbreaks (*48*–*53*) to the Pacific setting, on the basis of syndromic surveillance as the common surveillance system (Table 2) (*46*,*47*). After the issuance of these recommendations, the outbreak of CHIKV infection in Yap State was declared in August 2013 and reported in a timely manner on PacNet and elsewhere (*6*). The Yap State EpiNet team, the multidisciplinary national action team of PPHSN (*21*), has been effectively reporting weekly on PacNet on their efforts to map and control the outbreak. Regional assistance has been provided in the form of technical discussions and expertise in epidemiology and entomology from PPHSN partners. Furthermore, the Pacific Outbreak Manual (http://www.spc.int/phs/PPHSN/Surveillance/Syndromic/Pacific_Outbreak_Manual-version1-2.pdf) is being updated to include specific response guidelines for CHIKV outbreaks. PacNet has previously been shown to be sensitive in terms of number of messages on regional epidemics or on potential regional threats (*21*) and has continuously been used to update the region of the development of all the chikungunya outbreaks in PNG, New Caledonia, and Yap State.

**Table 2:** PPHSN recommendations for enhanced surveillance to the PICT in response to the threat of chikungunya outbreaks in the region

Category	Recommendations
Syndromic surveillance	We recommend enhanced surveillance with the purpose of prompt detection of any possible case in each PICT to organize a rapid response and mitigate the spread and impact as much as possible. National health authorities should ensure that their syndromic surveillance sentinel sites report weekly, adhere to case definitions, and report the number of patients that fit the case definitions of prolonged fever (any fever lasting ≥3 d) and AFR. These should be used as proxies for suspected chikungunya. Data on the other syndromes should be collected as usual. For countries/territories that use the optional syndrome dengue-like illness as part of their syndromic surveillance system, sentinel sites should report weekly also on this syndrome as it fits with chikungunya clinical symptoms. In addition to the existing surveillance system, any extension of the sentinel network or tracking trends at national level to detect chikungunya cases can be explored and discussed, depending on the local situation.
Vector control services to gear up in preparation	A review of supplies and equipment should be undertaken, orders placed if required, staff refreshed on community-based activities (larvicide) and spraying methods and protocols (adulticide).
Breeding sites elimination using awareness campaigns aimed at the general public	The time and season is appropriate to remind public about their role and responsibilities, and that the health authorities are intensifying relevant surveillance and response mechanisms as well. The campaigns do not have to focus on the risk specifically related to chikungunya but aim at vector control for vector-borne diseases, such as dengue and chikungunya, with a particular focus on container-breeding mosquitoes. Previous studies have shown that community engagement in vector control activities will achieve the greatest impact on reduction of *Aedes* mosquito vector species. All high-impact media and social networks should be asked to contribute, including educational and religious authorities.
Laboratory confirmation	If there is an unexpected rise in the number of reported cases of prolonged fever, AFR, or dengue-like illness, confirmation of the diagnosis by laboratory testing is recommended. Specimens should be tested for dengue and chikungunya.
Strongly suspected exposure (e.g., travel history to an outbreak area) or confirmed case	This should launch an immediate response, including: • recording of information on the case(s), including age, sex, place of residence, travel history, identification of geographic cluster and mobilization of affected communities; • vector control activities at the community level, focused around the residence of suspected cases, should be undertaken to eliminate potential breeding sites, and reduce the number of natural and artificial water-filled container habitats that support the breeding of mosquitoes; • spraying with insecticide to kill adult mosquitoes in the areas where the case-patient(s) reside(s) and work(s). Procedures must be clearly laid out and planned for by vector control services; • using mosquito repellents on affected and exposed people to reduce the transmission of the disease; • keeping potentially viremic patients (within the first 5–7 d of the disease) under impregnated (ideally) mosquito nets if admitted to a health facility; • starting rapid and effective risk communication to inform the public (e.g., provision of information on the situation, how to protect themselves); • disseminating treatment guidelines to hospitals and clinics to reduce the risk of hemorrhagic complications.

*Further details are available in the Pacific Outbreak Manual on the PPHSN Web site (http://www.spc.int/phs/PPHSN/Surveillance/Syndromic/Pacific_Outbreak_Manual-version1-2.pdf). PPHSN, Pacific Public Health Surveillance Network; PICT, Pacific Island Countries and Territories; AFR, acute fever and rash.

## Conclusion

CHIKV has reached the Pacific, with current chikungunya outbreaks occurring in PNG, Yap State, and New Caledonia. The threat of further spread is high. A large chikungunya outbreak in the Pacific would have severe effects on health care systems and public health infrastructure and would potentially affect general functions of society, as did the epidemic in the Indian Ocean region (*7*). Learning from previous experience is of the utmost importance, and governments and leaders in the region need to act in a timely manner and ensure balanced communication campaigns to inform and update public health professionals of the situation. In a region where many countries and territories struggle to meet International Health Regulation requirements, the management of the current threat of CHIKV in the Pacific is likely to also have implications for other parts of the world (*33*).

## References

[1] Dupont-Rouzeyrol M, Caro V, Guillaumot L, Vazeille M, D’Ortenzio E, Thiberge JM, chikungunya virus and the mosquito vector *Aedes aegypti* in New Caledonia (South Pacific Region). Vector Borne Zoonotic Dis. 2012;12:1036–41. 10.1089/vbz.2011.0937 23167500

[2] Staples JE, Breiman RF, Powers AM. Chikungunya fever: an epidemiological review of a re-emerging infectious disease. Clin Infect Dis. 2009;49:942–8. 10.1086/605496 19663604

[3] Burt FJ, Rolph MS, Rulli NE, Mahalingam S, Heise MT. Chikungunya: a re-emerging virus. Lancet. 2012;379:662–71. 10.1016/S0140-6736(11)60281-X 22100854

[4] Horwood PF, Reimer LJ, Dagina R, Susapu M, Bande G, Katusele M, Outbreak of chikungunya virus infection, Vanimo, Papua New Guinea. Emerg Infect Dis. 2013;19:1535–8. 10.3201/eid1909.130130 23965757 PMC3810919

[5] Chikungunya (13): New Caledonia. ProMED. 2013 Apr 29 [cited 2013 Dec 3]. http://www.promedmail.org, archive no. 20130430.1681533.

[6] Chikungunya (47): Micronesia (Yap) alert. ProMED. 2013 Nov 11 [cited 2013 Dec 3]. http://www.promedmail.org, archive no. 20131114.2055640.

[7] Renault P, Solet JL, Sissoko D, Balleydier E, Larrieu S, Filleul L, A major epidemic of chikungunya virus infection on Reunion Island, France, 2005–2006. Am J Trop Med Hyg. 2007;77:727–31. 17978079

[8] Pastorino B, Muyembe-Tamfum JJ, Bessaud M, Tock F, Tolou H, Durand JP, Epidemic resurgence of chikungunya virus in Democratic Republic of the Congo: identification of a new central African strain. J Med Virol. 2004;74:277–82. 10.1002/jmv.20168 15332277

[9] Porter KR, Tan R, Istary Y, Suharyono W, Sutaryo, Widjaja S, A serological study of chikungunya virus transmission in Yogyakarta, Indonesia: evidence for the first outbreak since 1982. Southeast Asian J Trop Med Public Health. 2004;35:408–15. 15691147

[10] Chretien JP, Anyamba A, Bedno SA, Breiman RF, Sang R, Sergon K, Drought-associated chikungunya emergence along coastal East Africa. Am J Trop Med Hyg. 2007;76:405–7. 17360859

[11] Chikungunya and dengue, south-west Indian Ocean. Wkly Epidemiol Rec. 2006;81:106–8. 16673456

[12] Renault P, Balleydier E, D’Ortenzio E, Baville M, Filleul L. Epidemiology of chikungunya infection on Reunion Island, Mayotte, and neighboring countries. Med Mal Infect. 2012;42:93–101. 10.1016/j.medmal.2011.12.002 22280563

[13] Chikungunya—Indian Ocean update (33): Maldives. ProMED. 2013 Dec 24 [cited 2012 Dec 3]. http://www.promedmail.org, archive no. 20061224.3598.

[14] Pulmanausahakul R, Roytrakul S, Auewarakul P, Smith DR. Chikungunya in Southeast Asia: understanding the emergence and finding solutions. Int J Infect Dis. 2011;15:e671–6. 10.1016/j.ijid.2011.06.002 21775183

[15] Outbreak and spread of chikungunya. Wkly Epidemiol Rec. 2007;82:409–15. 18035647

[16] Leo YS, Chow AL, Tan LK, Lye DC, Lin L, Ng LC. Chikungunya outbreak, Singapore, 2008. Emerg Infect Dis. 2009;15:836–7. 10.3201/eid1505.081390 19402989 PMC2687008

[17] Pongsiri P, Auksornkitti V, Theamboonlers A, Luplertlop N, Rianthavorn P, Poovorawan Y. Entire genome characterization of Chikungunya virus from the 2008–2009 outbreaks in Thailand. Trop Biomed. 2010;27:167–76. 20962712

[18] Jansen KA. The 2005–2007 chikungunya epidemic in reunion: ambiguous etiologies, memories, and meaning-making. Med Anthropol. 2013;32:174–89. 10.1080/01459740.2012.679981 23406067

[19] Alibert A, Pfannstiel A, Grangeon JP. Chikungunya outbreak in New Caledonia in 2011, Status report as at 22 August 2011. Inform’Action. 2011;34:3–9 [cited 2013 Dec 3] http://www.spc.int/phs/english/publications/informaction/IA34/Chikungunya_outbreak_New_Caledonia_status_report_22August2011.pdf

[20] Pavlin B. Chikungunya (10)—Papua New Guinea response. ProMED. 2013 Mar 24 [cited 2013 Dec 3. http://www.promedmail.org, archive no. 20130325.1602542.

[21] Souarès Y; Pacific Public Health Surveillance Network. Telehealth and outbreak prevention and control: the foundations and advances of the Pacific Public Health Surveillance Network. Pac Health Dialog. 2000;7:11–28. 11588911

[22] Horwood P, Bande G, Dagina R, Guillaumot L, Aaskov J, Pavlin B. The threat of chikungunya in Oceania. Western Pac Surveill Response J. 2013;4:8–10. 10.5365/wpsar.2013.4.2.003 24015365 PMC3762969

[23] Hide R. Chikungunya (12): Australia (Queensland) ex Papua New Guinea. 2013 Apr 27 [cited 2013 Dec 3]. http://www.promedmail.org, archive no. 20130427.1675486

[24] Queensland Health statewide weekly communicable diseases surveillance report, 25 November 2013 (for periods 18 November 2013–24 November 2013). 2013 Nov 25 [cited 2013 Dec 3]. http://www.health.qld.gov.au/ph/documents/cdb/weeklyrprt-131125.pdf

[25] Tsetsarkin KA, Vanlandingham DL, McGee CE, Higgs S. A single mutation in chikungunya virus affects vector specificity and epidemic potential. PLoS Pathog. 2007;3:e201. 10.1371/journal.ppat.0030201 18069894 PMC2134949

[26] Guillaumot L, Ofanoa R, Swillen L, Singh N, Bossin HC, Schaffner F. Distribution of *Aedes albopictus* (Diptera, Culicidae) in southwestern Pacific countries, with a first report from the Kingdom of Tonga. Parasit Vectors. 2012;5:247. 10.1186/1756-3305-5-247 23130961 PMC3497854

[27] Powers AM, Logue CH. Changing patterns of chikungunya virus: re-emergence of a zoonotic arbovirus. J Gen Virol. 2007;88:2363–77. 10.1099/vir.0.82858-0 17698645

[28] de Lamballerie X, Leroy E, Charrel RN, Ttsetsarkin K, Higgs S, Gould EA. Chikungunya virus adapts to tiger mosquito via evolutionary convergence: a sign of things to come? Virol J. 2008;5:33. 10.1186/1743-422X-5-33 18304328 PMC2266737

[29] Schuffenecker I, Iteman I, Michault A, Murri S, Frangeul L, Vaney MC, Genome microevolution of chikungunya viruses causing the Indian Ocean outbreak. PLoS Med. 2006;3:e263. 10.1371/journal.pmed.0030263 16700631 PMC1463904

[30] Suivi de la resistance des moustiques aux insecticides. Noumea (New Caledonia): Institut Pasteur de Nouvelle-Caledonie; 2011.

[31] Jones KE, Patel NG, Levy MA, Storeygard A, Balk D, Gittleman JL, Global trends in emerging infectious diseases. Nature. 2008;451:990–3. 10.1038/nature06536 18288193 PMC5960580

[32] Tatem AJ, Huang Z, Das A, Qi Q, Roth J, Qiu Y. Air travel and vector-borne disease movement. Parasitology. 2012;139:1816–30. 10.1017/S0031182012000352 22444826

[33] Chretien JP, Linthicum KJ. Chikungunya in Europe: what’s next? Lancet. 2007;370:1805–6. 10.1016/S0140-6736(07)61752-8 18061039

[34] Grandadam M, Caro V, Plumet S, Thiberge JM, Souares Y, Failloux AB, Chikungunya virus, southeastern France. Emerg Infect Dis. 2011;17:910–3. 10.3201/eid1705.101873 21529410 PMC3321794

[35] Kanamitsu M, Taniguchi K, Urasawa S, Ogata T, Wada Y, Wada Y, Geographic distribution of arbovirus antibodies in indigenous human populations in the Indo-Australian archipelago. Am J Trop Med Hyg. 1979;28:351–63. 453438 10.4269/ajtmh.1979.28.351

[36] Tesh RB, Gajdusek DC, Garruto RM, Cross JH, Rosen L. The distribution and prevalence of group A arbovirus neutralizing antibodies among human populations in Southeast Asia and the Pacific islands. Am J Trop Med Hyg. 1975;24:664–75. 1155702 10.4269/ajtmh.1975.24.664

[37] Borgherini G, Poubeau P, Staikowsky F, Lory M, Le Moullec N, Becquart JP, Outbreak of chikungunya on Reunion Island: early clinical and laboratory features in 157 adult patients. Clin Infect Dis. 2007;44:1401–7. 10.1086/517537 17479933

[38] Lagacherie P. Coverage of the chikungunya epidemic on Reunion Island in 2006 by the French healthcare system [in French]. Med Trop (Mars). 2012;72:97–8. 22693939

[39] Gérardin P, Fianu A, Malvy D, Mussard C, Boussaid K, Rollot O, Perceived morbidity and community burden of chikungunya in La Reunion [in French]. Med Trop (Mars). 2012;72:76–82. 22693934

[40] Flahault A, Aumont G, Boisson V, de Lamballerie X, Favier F, Fontenille D, Chikungunya, La Reunion and Mayotte, 2005–2006: an epidemic without a story? [in French]. Sante Publique. 2007;19(Suppl 3):S165–95. 17929405

[41] Malavankar DV, Puwar TI, Murtola TM, Vasan SS. Quantifying the impact of chikungunya and dengue on tourism revenues. Ahmedabad (India): Indian Institute of Management; 2009.

[42] Flahault A, Aumont G, Boisson V, de Lamballerie X, Favier F, Fontenille D, An interdisciplinary approach to controlling chikungunya outbreaks on French islands in the south-west Indian ocean. Med Trop (Mars). 2012;72:66–71. 22693932

[43] Schwartz O, Albert ML. Biology and pathogenesis of chikungunya virus. Nat Rev Microbiol. 2010;8:491–500. 10.1038/nrmicro2368 20551973

[44] Gaüzère BA, Gerardin P, Vandroux D, Aubry P. Chikungunya virus infection in the Indian Ocean: lessons learned and perspectives [in French]. Med Trop (Mars). 2012;72:6–12. 22693919

[45] Bâville M, Dehecq JS, Reilhes O, Margueron T, Polycarpe D, Filleul L. New vector control measures implemented between 2005 and 2011 on Reunion Island: lessons learned from chikungunya epidemic [in French]. Med Trop (Mars). 2012;72:43–6. 22693927

[46] Paterson BJ, Kool JL, Durrheim DN, Pavlin B. Sustaining surveillance: evaluating syndromic surveillance in the Pacific. Glob Public Health. 2012;7:682–94. 10.1080/17441692.2012.699713 22817479 PMC3457036

[47] Kool JL, Paterson B, Pavlin BI, Durrheim D, Musto J, Kolbe A. Pacific-wide simplified syndromic surveillance for early warning of outbreaks. Glob Public Health. 2012;7:670–81. 10.1080/17441692.2012.699536 22823595 PMC3419547

[48] Centers for Disease Control and Prevention, Pan American Health Organization. Preparedness and response for chikungunya virus introduction in the Americas. Washington (DC): The Organization; 2011.

[49] Institut National de Prévention et d’Education pour la Santé. Dossier spécial chikungunya—point sur les connaissances et la conduite à tenir. Saint-Denis (France): Institut National de Prévention et d’Education pour la Santé (INPES); 2008 [cited 2013 May 10]. http://www.inpes.sante.fr/CFESBases/catalogue/pdf/1085.pdf

[50] Programme de surveillance, d’alerte et de gestion du risque d’émergence du virus chikungunya dans les départements français d’Amérique. Prefecture de Martinique. Ministère de la Santé et des Soidarités—République Française; 2007 [cited 2013 May 10]. http://opac.invs.sante.fr/doc_num.php?explnum_id=3518

[51] World Health Organization Regional Office for South-East Asia. Guidelines on clinical management of chikungunya fever. 2008 [cited 2013 May 10]. http://www.wpro.who.int/mvp/topics/ntd/Clinical_Mgnt_Chikungunya_WHO_SEARO.pdf

[52] Abeyewickreme W, Wickremasinghe AR, Karunatilake K, Sommerfeld J, Axel K. Community mobilization and household level waste management for dengue vector control in Gampaha district of Sri Lanka; an intervention study. Pathog Glob Health. 2012;106:479–87. 10.1179/2047773212Y.0000000060 23318240 PMC3541909

[53] Arunachalam N, Tyagi BK, Samuel M, Krishnamoorthi R, Manavalan R, Tewari SC, Community-based control of *Aedes aegypti* by adoption of eco-health methods in Chennai City, India. Pathog Glob Health. 2012;106:488–96. 10.1179/2047773212Y.0000000056 23318241 PMC3541894

